# Elucidating the Functional Roles of Long Non-Coding RNAs in Alzheimer’s Disease

**DOI:** 10.3390/ijms25179211

**Published:** 2024-08-25

**Authors:** Zhenyu Huang, Qiufen Chen, Xuechen Mu, Zheng An, Ying Xu

**Affiliations:** 1 College of Computer Science and Technology, Jilin University, Changchun 130012, China; zhenyuh19@mails.jlu.edu.cn; 2 Systems Biology Lab for Metabolic Reprogramming, Department of Human Genetics and Cell Biology, School of Medicine, Southern University of Science and Technology, Shenzhen 518055, China; chenqf829@foxmail.com (Q.C.); m250921296@gmail.com (X.M.); 3 School of Mathematics, Jilin University, Changchun 130012, China; 4 School of Medicine, Indiana University, Indianapolis, IN 46202, USA; anz@iu.edu

**Keywords:** Alzheimer’s disease, long non-coding RNAs, oxidative stress, Fenton reaction

## Abstract

Alzheimer’s disease (AD) is a multifaceted neurodegenerative disorder characterized by cognitive decline and neuronal loss, representing a most challenging health issue. We present a computational analysis of transcriptomic data of AD tissues vs. healthy controls, focused on the elucidation of functional roles played by long non-coding RNAs (lncRNAs) throughout the AD progression. We first assembled our own lncRNA transcripts from the raw RNA-Seq data generated from 527 samples of the dorsolateral prefrontal cortex, resulting in the identification of 31,574 novel lncRNA genes. Based on co-expression analyses between mRNAs and lncRNAs, a co-expression network was constructed. Maximal subnetworks with dense connections were identified as functional clusters. Pathway enrichment analyses were conducted over mRNAs and lncRNAs in each cluster, which served as the basis for the inference of functional roles played by lncRNAs involved in each of the key steps in an AD development model that we have previously built based on transcriptomic data of protein-encoding genes. Detailed information is presented about the functional roles of lncRNAs in activities related to stress response, reprogrammed metabolism, cell polarity, and development. Our analyses also revealed that lncRNAs have the discerning power to distinguish between AD samples of each stage and healthy controls. This study represents the first of its kind.

## 1. Introduction

Alzheimer’s disease (AD) is a complex disease with numerous pathological changes, such as altered homeostasis of extra- and intracellular pH [1,2], disrupted equilibrium of crucial intracellular ions such as $N_{a}^{+}$ and $K^{+}$ [3,4], chronic inflammation, heightened oxidative stress [5,6], elevated neurotoxicity, and extensive neuronal apoptosis [7,8]. While considerable attention has been devoted to unraveling the causes and consequences of amyloid-beta (A*β*) plaque development and Tau-based neurofibrillary tangle (NFT) formation in the past three decades, recent studies have raised doubts regarding their proposed central roles in the reduced cognitive capacities in AD patients [9]. Instead, a preponderance of evidence suggests extensive neuronal death in specific cerebral regions as the key determinant of the cognitive decline in the affected individuals [10]. However, with great amounts of omics data and information amassed in the field of AD research, disentangling the intricate causal relationships among these pathological conditions and their relevance to extensive neuronal loss remains a great challenge. Numerous hypotheses have been put forward regarding the drivers and key mechanisms of the development of AD, including oxidative stress in mitochondria [11] and dysregulated intracellular pH, which can impair the functions of acidic organelles like endosomes and lysosomes, becoming detrimental to the host neurons [12,13]. Additionally, the formation of Tau aggregates can precipitate cell death [14], while overexpression of the amyloid precursor protein (APP) may contribute to A*β*-associated neuronal death in advanced AD tissues [14].

Like in studies of other human diseases, the vast majority of the published work on AD has been protein or protein–gene-centric [15], while the roles played by long non-coding RNAs (lncRNAs) have been largely un- or under-explored [13]. Limited knowledge about lncRNAs in the pathogenesis of AD includes those involved in amyloid formation [16], Tau protein hyperphosphorylation [17], and the oxidative stress response [18]. As a comparison, the functional roles played by lncRNAs under physiological conditions have been extensively studied in brain development, homeostasis, the oxidative stress response, plasticity, and evolution [11,12,13]. It has been well established that 40% of the human lncRNA genes are expressed in the brain. Hence, there should be no surprise if lncRNAs play important roles in AD pathogenesis.

In this study, we have performed transcript-level assembly of all RNAs from raw RNA-seq data collected from the prefrontal cortex tissues of both healthy individuals and AD patients, which are publicly available [19]. The reason for performing our own assembly of transcripts is that the available transcripts of RNA genes in AD tissues in the public domain are far from adequate for meaningful analyses. Specifically, transcripts for only 11,300 lncRNA genes have been detected in AD tissues and made publicly available, which is clearly too low, knowing that overall, 228,048 transcripts for 48,479 lncRNA genes have been detected in healthy human tissues and publicly available. Our assembly resulted in 431,781 transcripts for 55,098 lncRNA genes, having considerably expanded the numbers of both the transcripts and the genes.

**Figure 1:**
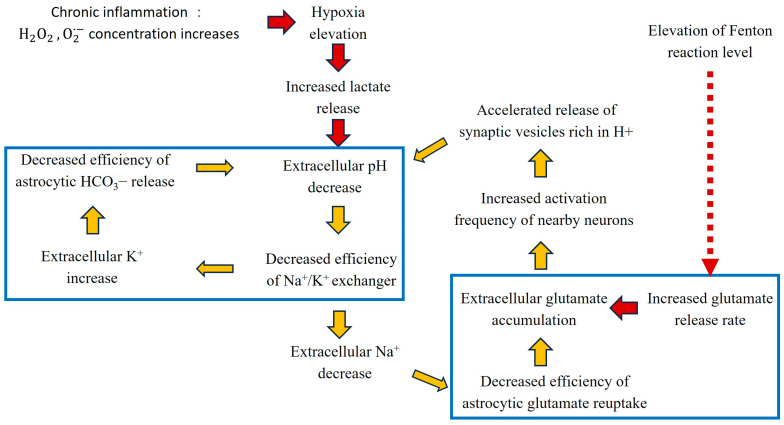
A model for two vicious cycles driven by Fenton reactions throughout the progression of AD. The yellow arrows indicate the phenotypes of the vicious cycles. The red arrows represent the phenotypes that drive these vicious cycles.

It has been well established that AD tissue cells have increased intracellular pH with a pH of roughly 7.037 and a decreased extracellular pH of 6.85, compared to the normal ones of ~7.028 [20] and 6.9 [21,22]. We have conducted a computational analysis of the transcriptomic data of AD tissues vs. controls, coupled with computational chemistry analyses, to address two main questions: (1) what are the causes and consequences of AD cell intracellular alkalization? and (2) what are the causes and consequences of AD cell extracellular acidification? Our answers to these questions naturally give rise to a model for AD formation and development (manuscript under review), consisting of the following key steps and illustrated in Figure 1:

(1) Chronic inflammation (chemically, the levels of $H_{2}O_{2}$ and $O_{2}^{\cdot -}$ are significantly increased) coupled with iron and/or copper accumulation leads to persistent Fenton reactions in mitochondria:

$$O_{2}^{\cdot -}+H_{2}O_{2}\overset{F_{e}^{2+}}{\to }\cdot OH+{OH}^{-}+O_{2}\;\mathrm{or}\;O_{2}^{\cdot -}+H_{2}O_{2}\overset{C_{u}^{+}}{\to }\cdot OH+{OH}^{-}+O_{2}.$$

(2) Mitochondrial persistent Fenton reactions drive the pH up as reflected by the increased expressions of the established marker genes CFTR, CLN3, and NOX1 [23], leading to cell death if not neutralized; (3) multiple acidifying metabolic reprograms are induced to produce H^+^ to keep the pH stable, with the key ones being hydrolysis of glutamine to glutamate and N$H_{4}^{+}$, catalyzed by GLS,

$$\mathrm{Gln}+H_{2}O\;\overset{GLS}{\to }\;\mathrm{Glu}+NH_{3}+H^{+},$$

and hyperphosphorylation of the Tau proteins bound with microtubules, which produces one H^+^ per phosphorylation, resulting in an acidic Tau fiber structure [24]. (4) Persistently produced Glu are released into the extracellular space, reflected by increased expression of the transporter gene SLC25A22 [25] from mitochondria to the cytosol, as well as the transporter gene SLC17A6 [26] from the cytosol to the extracellular space and their co-expressions with the predicted level of mitochondrial Fenton reactions. (5) The over-produced and released glutamates drive the neighboring neurons to become hyperexcited, as reflected by the increased expression of the established marker genes CNTNAP2 and KCNQ2 [27]. This gives rise to the over-production and release of acidic synaptic vesicles, as reflected by the increased expression of the established marker genes BCL2L1 and ACTG1 [28] resulting in acidification of the extracellular space, reflected by the increased expression of marker genes ASIC2 and ASIC3 [29]. (6) Under physiological conditions, the H^+^ released by synaptic vesicles will be neutralized by bicarbonates released by nearby astrocytes. While under the condition of increasing extracellular acidification, the release rate of bicarbonate by astrocytes will not keep up with the release rate of synaptic vesicles, as reflected by the expressions of the relevant marker genes SLC4A1, SLC4A2, and SLC4A3 [30], forming one vicious cycle involving increased extracellular K^+^ levels and further decreased extracellular pH. (7) As the disease evolves, the extracellular level of glutamate accumulation increases, reflected by marker genes SLC1A2 and SLC1A3’s expression. This is a result of a second vicious cycle involving decreased extracellular Na^+^ levels, further decreased extracellular pH, and a reduced ability to clear extracellular glutamates. (8) As a key response to progressively increasing extracellular acidification, cells increase their release rate of $C_{u}^{+}$, resulting in an extracellular Fenton reaction,

$$O_{2}^{\cdot -}+H_{2}O_{2}\overset{C_{u}^{+}}{\to }\cdot OH+{OH}^{-}+O_{2},$$

which helps to slow down extracellular acidification in two ways: the persistent production of ${OH}^{-}$ and the formation of alkaline Aβ plaques [31] as the result of interactions between OH and Aβ monomers [32]. It is noteworthy that the model is predicted based on the following information: (1) altered expression of the relevant marker genes; (2) strong correlations between marker genes’ expression in consecutive steps in the model; and (3) balanced production and consumption of H^+^s in key steps across the entire model.

Our analyses have revealed that extracellular acidosis represents the leading cause of neuronal cell death in AD, followed by the formation of Aβ plaques, only in advanced stage AD, while it plays a role in slowing down extracellular acidosis in the early phase of the disease (manuscript under review).

Compared to our above work, this study focuses on the functional roles played by lncRNAs throughout the disease development of AD, adding a missing component from our previous model. Here, we have developed an ordering of the disease samples based on, in essence, the distance between each disease sample and the control samples in terms of the expression data of metabolic genes known to be relevant to the disease, aiming to reflect the progression of the disease, called a pseudo-time course. Using this ordered sample list, we have made the following observations regarding lncRNA gene involvement in AD pathogenesis:

1. Expressed lncRNAs are primarily involved in the upregulated functions in AD tissues, ranging from immune activities and metabolism to cell polarity and stress responses, which seem to play driving roles in the disease progression, while only a few lncRNAs are involved in downregulated functions.

2. The most enriched pathways by lncRNAs expressed in AD tissues are relevant to oxidative stress.

3. LncRNA-mediated reprogrammed metabolism is involved in alleviating intracellular alkaline stress and extracellular acidic stress.

4. LncRNAs as biomarkers have strong predictive power in distinguishing (early-stage) AD patients from healthy controls.

To the best of our knowledge, this is the first large-scale analysis of the functional roles played by lncRNAs throughout AD development.

## 2. Results

### 2.1. Identification and Characterization of Novel lncRNAs

We have assembled the transcriptome from raw RNA-seq data from 527 AD and control tissue samples and then removed transcripts for protein genes (Data and Methods). This resulted in 55,098 predicted lncRNAs. The clinical information of these samples is summarized in Supplementary Table S1.

**Figure 2:**
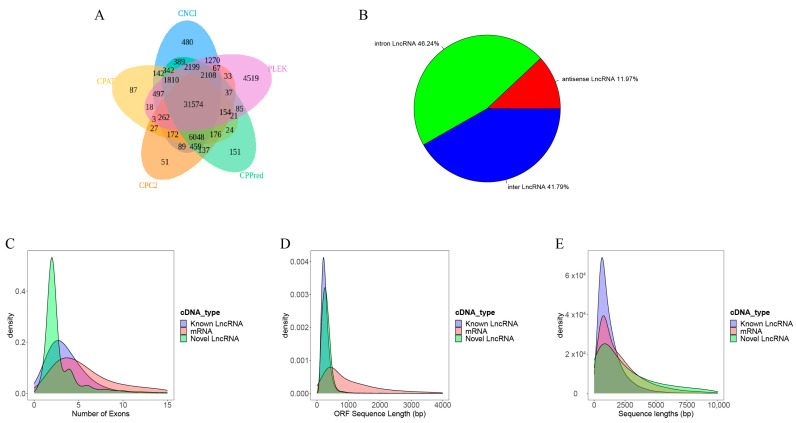
Prediction of novel lncRNAs in AD tissues. (**A**) Venn analysis of the predicted novel lncRNAs using five softwares:CNCI v1.0, CPC v0.1, CPPred v1, PLEK V1.2, and CAPT v2.0.0. (**B**) Classification of predicted novel lncRNAs. (**C**) Exon numbers in protein-coding genes, known lncRNA genes, and novel lncRNAs. (**D**) ORF length distributions of protein-coding genes, known lncRNAs, and novel lncRNAs. (**E**) Sequence lengths of protein-coding genes, known lncRNAs, and novel lncRNAs.

Of these lncRNAs, 31,574 are of high quality and novel, namely not present in the GENCODE database [33], as summarized in Figure 2A and detailed in Supplementary Data S1. A total of 41.79% of these transcripts were in intergenic regions, 46.24% in the intronic regions, and 11.97% were the antisense of protein-encoding genes (Figure 2B), suggesting their possible roles in transcriptional regulation of the relevant protein genes.

Previous studies have shown that lncRNAs in mammals are shorter in length and have fewer exons than protein-coding genes [27,28,29]. To examine whether the lncRNAs expressed in AD samples have the same characteristics, we have compared the structures of our assembled lncRNA transcripts (Figure 2C–E) using the two-sided Wilcoxon test [34,35]. We note that the lnRNAs tend to have considerably shorter average lengths than mRNAs (1521 bp vs. 1842 bp with *p*-value < 2.22 × 10^−16^, as shown in Figure 2C and Figure S1). They also have shorter open reading frames (ORFs) compared to protein-coding genes (279 bp vs. 759 bp, adjusted *p*-value < 2.22 × 10^−16^, Figure 2D and Figure S1) and are encoded by fewer exons (two vs. five, *p*-value < 2.22 × 10^−16^, Figure 2E and Figure S1).

### 2.2. LncRNAs’ Roles in AD Formation and Development

**Figure 3:**
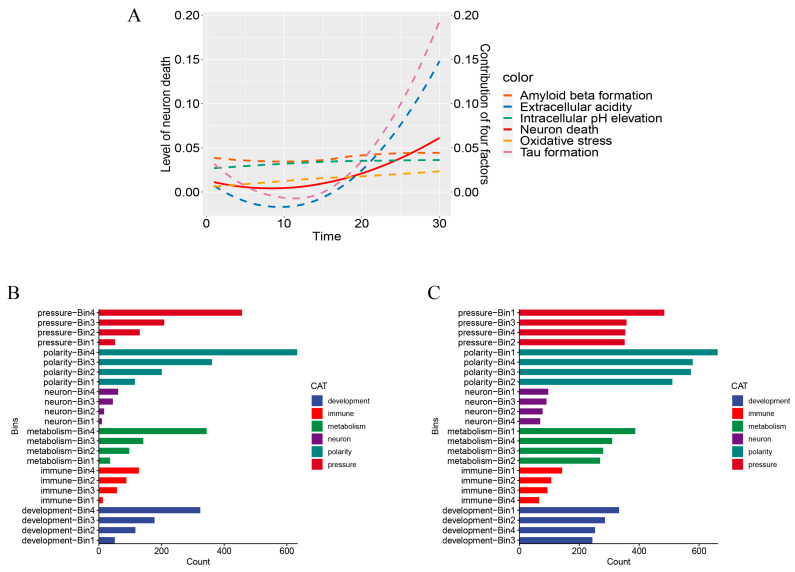
Statistics related to the pathological progression of lncRNAs in AD. (**A**) The relative changes in the levels of A*β* plaques, Tau fibers, intracellular alkalinity, extracellular acidity, oxidative stress, and apoptosis compared to controls throughout the disease progression. (**B**) Category statistics of different upregulated pathways in different bins. (**C**) Category statistics of different downregulated pathways in different bins.

To study how the disease phenotypes change with the progression of AD, we constructed an ordering of the AD samples, aiming to capture the progression of the disease (see Section 4.2). We then partitioned the list of samples into four equal-sized sub-lists, bin1, bin2, bin3, and bin4, possibly with fewer samples in the last sub-list, bin4 (Figure 3A). Across bins 1 to 4, we observed a significant increase in AD cell amyloid plaques, Tau fibers, and neuronal death, while extracellular acidity, intracellular alkalinity stress, and oxidative stress rose gradually. We then constructed an lncRNA–mRNA co-expression network over samples within each bin (see Section 4.2), resulting in 45, 54, 47, and 35 clusters in the four bins, respectively. This was followed by a pathway enrichment analysis over samples in each bin, resulting in a total of 8000 distinct pathways.

We grouped these pathways into six categories: development-related, immune activity, metabolism, neural function, cell polarity, and stress response. The detailed sets of these six categorized pathways can be found in Supplementary Data S2. We noted the following: (1) the number of downregulated pathways in each category remained generally stable across the four bins, namely throughout the progression of AD; (2) the numbers of upregulated pathways in the categories of development-related, immune activity, metabolism, cell polarity, and stress response increased with the disease progression, while those in neural function remained relatively stable (Figure 3B,C). Most of the developmental pathways consisted of muscle development, cell morphology, and a few others. Consequently, we hypothesize that lncRNAs are primarily involved in cell polarity, stress response, immunity, and metabolism in AD. In the following, we investigate how lncRNAs may involve these pathways throughout the AD progression.

**Figure 4:**
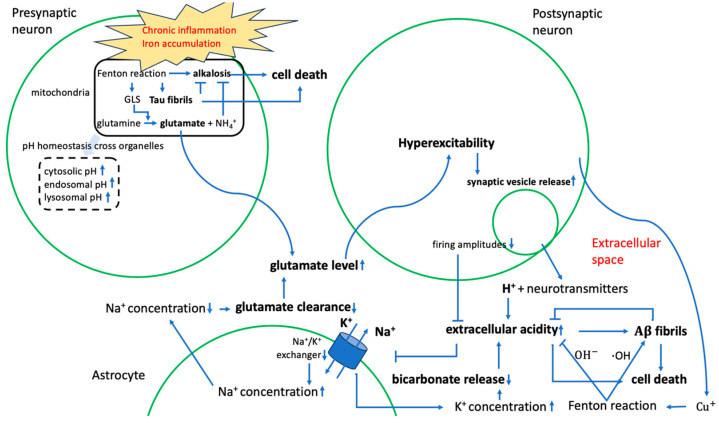
A schematic of our model, composed of key events and logical relationships, where each arrow represents a causal relationship.

Figure 4 summarizes the key components of our protein-centric model for AD development (manuscript under review). The detailed gene sets for each step can be found in Supplementary Data S3. Here, we study how lncRNAs contribute to the key components in this model as the disease progresses from bin1 to bin4. We quantified the level of contribution by each pathway to AD progression from bin1 to bin4, where the level of contribution is defined using the discerning score by each feature (see Section 4.2).

#### 2.2.1. Contributions to Key AD Phenotypes via Cell Polarity Changes

**Figure 5:**
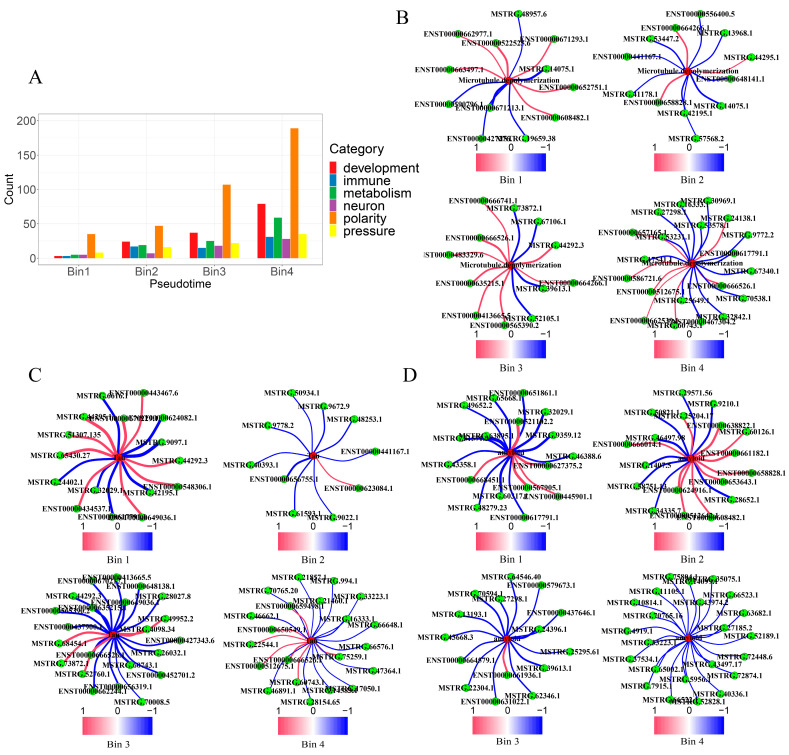
LncRNAs contributing to key AD phenotypes through cell polarity changes. (**A**) Statistics of the top 20% of pathways for each group of pathways in each bin. (**B**) The regulatory trends of lncRNAs associated with microtubule depolymerization from bin1 to bin4. Blue nodes represent inhibition, while red nodes are for promotion. Central nodes are gene-set names, surrounded by green nodes representing regulatory lncRNAs. Similar regulatory diagrams follow the same plotting logic. (**C**) The numbers of lncRNAs involved in Tau fiber formation across the four bins. (**D**) The numbers of lncRNAs involved in A*β* plaque formation across the four bins.

We note that cell polarity-related pathways consistently contributed the highest levels of AD progression across all four bins (Figure 5A and Figure S2, Table S2). The most contributing cell polarity pathways were filament-bundle assembly, microtubule polymerization or depolymerization, metal ion transport, and transport of acidic organic compounds (Table S2).

In AD, filament bundle assembly refers to the formation of intracellular Tau fibril bundles and the formation of extracellular A*β* plaques [14]. The process of intracellular Tau fiber formation starts from the disassociation of the Tau proteins and microtubules that they bind, resulting in free Tau monomers in the intracellular space and then the formation of Tau aggregates.

We noted that the number of lncRNAs involved in microtubule depolymerization via freeing up Tau proteins were 14, 5, 208, and 185 from bin1 through bin4, respectively (Figure 5B, Table S3). Examples include lncRNA ENST00000666526.1-chr2:65047854-65056433, which enhances the transcription of KIFC3, which is involved in microtubule transport and positioning [36], and STMN1, which promotes microtubule depolymerization [37]. Interestingly, lncRNAs were also involved in inhibiting microtubule depolymerization, with the number of such lncRNAs being 18, 30, 278, and 3387 across the four bins, respectively (Figure 5B, Table S3). One example is lncRNA MSTRG.54738.1-chr4:184435028-184467535, involved in suppressing the expressions of STMN1 and SPAST, where the latter is a microtubule-severing protein involved in regulating microtubule dynamics (Table S3) [38]. We postulate that the differences in the numbers of lncRNAs involved in promoting vs. those in inhibiting microtubule depolymerization reflect a shift in the homeostasis of cellular populations of microtubules.

We also noted that the number of lncRNAs involved in Tau-fiber formation were 17, 11, 170, and 182 from bin1 to bin4, while the number of those in inhibiting Tau-fiber formation was 21, 137, 175, and 3056, respectively (Figure 5C, Table S4). For example, lncRNA MSTRG.44295.1-chr20:63431768-63433141 exhibits the strongest promotion of BRSK2 transcripts across the four bins (Table S4). Interestingly, BRSK2 is also heavily suppressed by lncRNAs, with MSTRG.61027.1-chr6:75606827-75610921 showing the greatest inhibitory potency (Table S4). Again, these data suggest that there are opposing forces in promoting and inhibiting the formation of Tau fibers, which is consistent with published studies [39] and our own data (Figure 5C and Figure S3). To further understand this, a pathway-enrichment analysis over mRNAs correlated with lncRNAs involved in Tau fiber formation, as well as with lncRNAs in inhibiting its formation. The results show that (1) pathways correlated with Tau-fiber formation are predominantly involved in the intracellular alkaline-stress response, and (2) pathways relevant to inhibiting Tau fibers are neuronal death (Table S4).

Similar observations are made about lncRNA involvement in the development of A*β* plaques. Specifically, in the early-stage samples (bin1 and bin2), lncRNAs were mostly involved in the promotion of A*β* plaque formation, while in the advanced-stage samples (bin3, bin4), lncRNAs were heavily involved in the inhibition of A*β* plaque formation (Figure 5D). Examples include (1) the lncRNAs in Table S5 involved in expressing CLU, a protein crucial for A*β* plaque formation, and (2) lncRNA MSTRG.19468.1-chr13:99195282-99291580, involved in inhibiting A*β* plaque formation (Table S5). Our explanation is that the formation of A*β* plaques plays a role in slowing down the extracellular acidosis process, as the copper-mediated extracellular Fenton reactions produce OH^−^ and the alkaline A*β* plaque structures as outlined in the Introduction, while with the increase in both the density and sizes of A*β* plaques, they become increasingly toxic to the nearby neurons [31].

#### 2.2.2. Contribution to Stress Generation

The most significant stressors throughout AD development are intracellular alkalization and extracellular acidification, both being the results of mitochondrial Fenton reactions (Figure 4 and manuscript under review). Our previous study [40] revealed that AD involves at least two classes of Fenton reactions, one in mitochondria, either iron or copper-mediated, which we consider as an initial driver of the formation of AD, and one in the extracellular space, predominantly copper-mediated, which serves a positive role in slowing-down extracellular acidosis through the formation of A*β* plaques that become deadly as they continue to accumulate [41].

**Figure 6:**
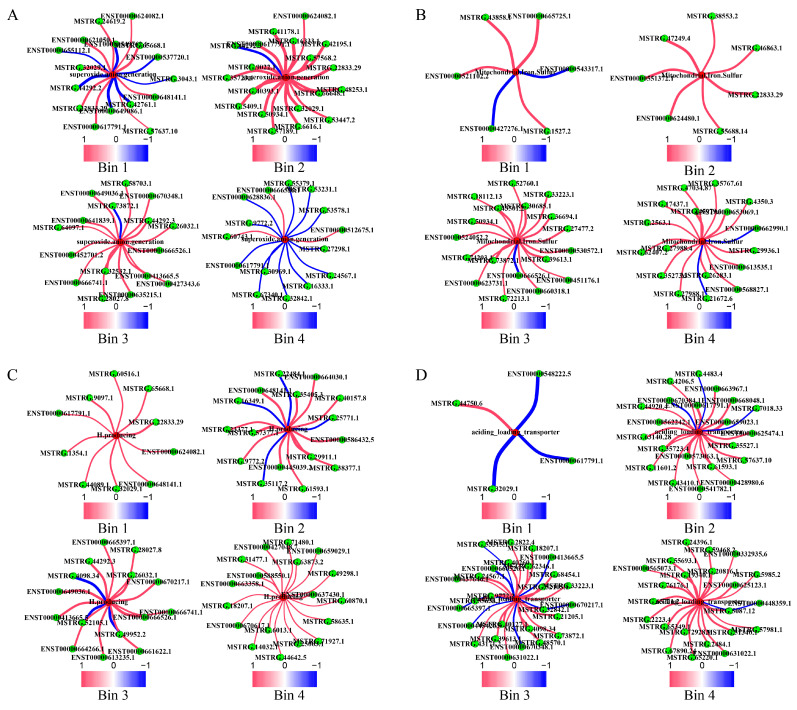
LncRNAs involved in the induction of metabolic reprogramming for relieving stresses. (**A**) The numbers of lncRNAs involved in superoxide anion generation across the four bins. (**B**) The numbers of lncRNAs involved in mitochondrial iron–sulfur clustering synthesis across the four bins. (**C**) The numbers of lncRNAs involved in H^+^-producing enzymes across the four bins. (**D**) The numbers of lncRNAs involved in acid loading transporter across the four bins.

The basis for Fenton reactions is the over-production and accumulation of $H_{2}O_{2}$ and ${\cdot O}_{2}^{-}$ by local astrocytes. LncRNAs are involved in the promotion of $H_{2}O_{2}$ and ${\cdot O}_{2}^{-}$ production in bin1 and bin2 and in the inhibition of their production in bin3 and bin4 (Figure 6A and Figure S4). The key proteins involved in $H_{2}O_{2}$ production are COX2, SOD2, and MFN2. LncRNA ENST00000560481.2-chr1:150965245-150966130 is a key promoter of the expressions of these proteins, while lncRNA ENST00000667795.1-chr12:126165730-126192674 is a key inhibitor (Table S6). Similarly, the key proteins involved in ${\cdot O}_{2}^{-}$ production are APP, GNAI2, and MAPT, with lncRNA ENST00000666526.1-chr2:65047854-65056433 being a key promoter (Table S6). HVCN1, AGT, MAPT, CYBA, and APP are involved in inhibiting the production of ${\cdot O}_{2}^{-}$, with lncRNA MSTRG.16333.1-chr12:78881520-78886563 being a key inhibitor (Table S6).

Regarding the roles played by lncRNAs in mitochondrial iron and copper transport, we noted that the number of lncRNAs involved in iron accumulation in mitochondria was 4, 7, 48, and 54, respectively, while those involved in inhibitory roles were 2, 0, 16, and 5, respectively (Figure 6B, Table S7). Key relevant lncRNAs included MSTRG.45979.1-chr22:25181907-25218915 for promoting iron/copper accumulation and MSTRG.21672.6-chr14:79254858-79260292 for inhibiting such accumulation (Figure 6B, Table S7).

In addition, lncRNAs were also involved in promoting extracellular copper release, with their numbers being 113, 29, 260, and 173 from bin1 to bin4, while the numbers of lncRNAs involved in copper release were 63, 354, 360, and 3357 from bin1 to bin4, respectively (Figure S4, Table S8). Among these, MSTRG.24138.1-chr15:67418775-67526417 and MSTRG.16333.1-chr12:78881520-78886563 are examples, one for promoting and the other for inhibiting copper release (Figure S4, Table S8).

Synapse assembly, driven by neuronal firing triggered by extracellular glutamate, leads to the release of H^+^-rich synaptic vesicles, a key contributor to extracellular acidosis (see Introduction) (Figure S4, Table S9). LncRNAs such as MSTRG.43821.1-chr20:47106756-47108522 are predominantly involved in inhibiting synaptic assembly (Table S9).

#### 2.2.3. Contribution to Stress Response via Metabolic Reprogramming

Numerous reprogrammed metabolisms are induced in AD tissue cells, responding to intracellular alkalization or extracellular acidification. One class of reprogrammed metabolism, in which lncRNAs are heavily involved, is lipid metabolism, particularly cholesterol metabolism (see Table S2). Specifically, the numbers of lncRNAs involved in promoting cholesterol synthesis were 10, 10, 210, and 246 from bin1 through bin4, respectively, while the numbers of lncRNAs involved in inhibitory roles of cholesterol biosynthesis and metabolism were 14, 60, 245, and 3644 from bin1 through bin4, respectively (Figure S4, Table S10). This is not surprising, knowing that cholesterols have both pro- and anti-inflammatory functions [42].

Based on our model outlined in Section 2.2, we would expect to see AD cells are stressed with persistent intracellular alkalinity; we have analyzed all 1258 enzymes, each catalyzing H^+^-producing reactions [43]. A principal component analysis was conducted over these enzymes across all AD samples under study. Remarkably, the first principal component, or PC1, over samples in bin4 could explain 97.8% of the variance in the gene expression of these enzymes (Figure S5), strongly suggesting their consistent roles in alleviating intracellular alkaline stress. LncRNAs are involved in promoting H^+^-generating enzymes from bin1 to bin4, accounting for 32, 15, 188, and 95 lncRNAs, respectively, while the numbers of lncRNAs involved in inhibiting these enzymes were 5, 17, 205, and 33 from bin1 to bin4 (Figure 6C, Table S11). Among the top 5% of H^+^-producing enzymes contributing to PC1 in each bin, kinases dominated (Table S12). Glutaminase (GLS) was among these enzymes consistently involved in mitigating the alkalizing stress caused by mitochondrial Fenton reactions throughout all bins. Our study found that 50% of the lncRNAs that promote the production of H^+^ enzymes were involved in promoting GLS-related transcripts, such as ENST00000479552.1-chr2:190880872-190898209 (Table S12).

In addition, the number of lncRNAs involved in promoting transporters that acidify the intracellular space (Figure 6D) were 3, 28, 182, and 3308 from bin1 to bin4, while the number of lncRNAs involved in inhibiting such transporters were 8, 2, 91, and 136 (Table S13), suggesting that the balance is shifted towards producing more acidic molecules.

Table 1 lists the number of lncRNAs with high discerning scores involved in immune activity, metabolic reprogramming, cell polarity, and stress response pathways from bin1 to bin4. Supplementary Table S14 provides the full set of genes regulated by these lncRNAs.

### 2.3. LncRNA’s Discerning Power in Distinguishing AD Samples from Controls

**Figure 7:**
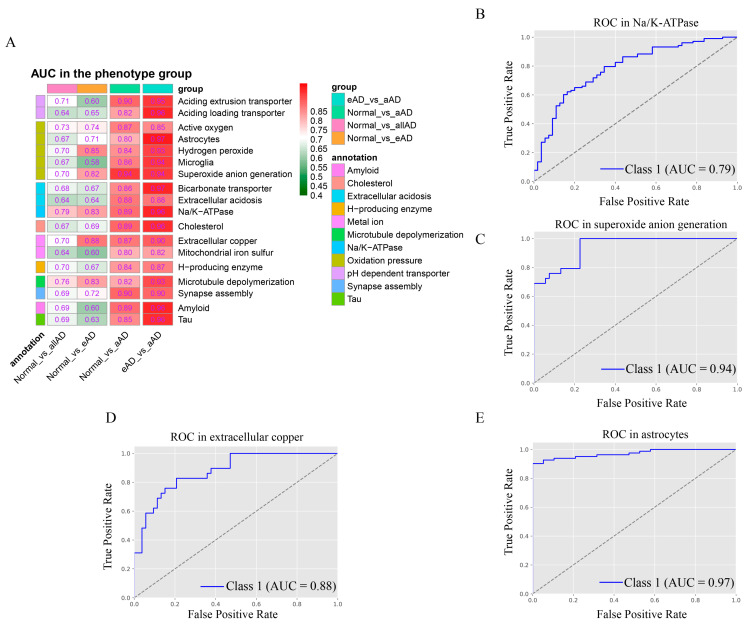
Assessment of lncRNAs’ discerning power between AD and control samples. (**A**) AUC scores by lncRNAs in distinguishing all AD samples vs. controls, late-stage AD samples vs. controls, earliest-stage AD samples vs. controls, and early-stage AD samples vs. mid-late-stage AD samples. (**B**) ROC curves by lncRNAs involved in Na^+^/K^+^-ATPase in distinguishing all AD samples vs. controls. (**C**) ROC curves by lncRNAs involved in regulating superoxide anion generation in differentiating late-stage AD samples vs. controls. (**D**) ROC curves by lncRNAs regulating extracellular copper in discriminating earliest-stage AD samples vs. controls. (**E**) ROC curves by lncRNAs involved in regulating astrocytes in distinguishing early-stage AD samples vs. mid-late-stage AD samples.

The above analyses revealed that lncRNAs are involved in the regulation of at least 22 pathways strongly associated with AD phenotypes, as shown in Figure 7A and Table 2. We aim to identify a subset of these lncRNAs that can distinguish between normal and AD tissues (or tissues of a specific stage of AD) well and which could potentially be used as blood biomarkers for AD detection. It is noteworthy that compared to proteins, lncRNAs tend to have longer half-lives in blood circulation as they tend to be more resistant to degradation [44,45,46].

Among the lncRNA-containing pathways, lncRNAs involved in regulating Na^+^/K^+^-ATPase, suppressed by extracellular acidosis [47], exhibit the best performance in distinguishing control from AD samples, with an AUC of 0.7866 (Figure 7B, Table 2), which is significantly better than an AUC of 0.655 achieved by CSF A*β*40/42 prediction performed by other authors [48]. In distinguishing normal from late-stage AD samples, the lncRNAs involved in regulating superoxide anion generation performed the best, with an AUC of 0.944 (Figure 7C, Table 2), considerably better than an AUC of 0.853 achieved by plasma p-tau181 prediction in an earlier study [49].

Furthermore, in distinguishing normal from the earliest stage AD samples, lncRNAs involved in regulating extracellular copper achieved a performance level at an AUC of 0.8783 (Figure 7D, Table 2). In distinguishing the earliest-stage from the late-stage AD samples, the lncRNAs involved in regulating astrocytes achieved an AUC of 0.9718 (Figure 7E, Table 2). Detailed information on these lncRNAs can be found in Table S15.

## 3. Concluding Remarks

We have conducted a computational analysis of the functional roles played by lncRNAs throughout the development of an AD. As the first step of this analysis, we have conducted transcript-level assembly from the raw RNA-seq data, resulting in a total of 55,098 lncRNAs, with 30,102 being novel.

The basis of our functional analyses of lncRNAs is the pathways enriched by mRNAs having known functional annotations, which are strongly co-expressed with the lncRNAs, coupled with their predicted functions as *cis* or *trans* regulators.

To elucidate how the functional roles played by lncRNAs change with the progression of the disease, we have defined a distance between the AD samples and the controls, which gives rise to an ordered list of AD samples from the early to the advanced stage. This provides an approximation to the disease progression. Our overall discovery can be summarized as follows:

1. Across all four bins representing different stages of AD progression, lncRNAs involved in cell polarity-related pathways consistently contributed significantly to AD progression. Notably, filament-bundle assembly, microtubule polymerization or depolymerization, metal ion transport, and transport of acidic organic compounds were among the most contributing pathways, for each of which lncRNAs are actively involved throughout AD progression.

2. Intracellular alkalization, extracellular acidification, and oxidative stress are major stressors and significantly contribute to AD progression. LncRNAs were involved in both stress generation and the stress responses of all three stressors, including iron and copper accumulation, as well as the formation of Tau fibers and A*β* plaques.

3. Intracellular alkalization and extracellular acidification induce multiple metabolic reprogramming in AD cells for survival. LncRNAs were involved in activating a majority of these reprogrammed metabolisms.

4. Figure 4 outlines the steps in our AD model, and lncRNAs were involved in most of these steps.

5. LncRNAs have strong discerning power in distinguishing AD samples from the controls, as well as in distinguishing AD samples of specific stages from the controls.

It should be noted that our study is performed on poly-A selected RNA-seq data, and hence, all results reported here may not be applicable to non-polyadenylated lncRNAs. We aim to include non-polyadenylated RNAs in our future study.

Overall, this study significantly expands our understanding of how lncRNAs participate in the progression of AD for the first time. The identification and characterization of novel lncRNAs provide valuable insights into their functional roles. The pseudo-time course provided highly useful information regarding how various phenotypes of AD change as the disease progresses. These findings may have significant implications for the development of diagnostic biomarkers and therapeutic targets for AD, paving the way for new avenues in research on lncRNA-based interventions for neurodegenerative diseases.

## 4. Materials and Methods

### 4.1. Data

Gene expression data: The raw reads of 529 samples were downloaded from the ROSMAP cohort (https://www.synapse.org/Synapse:syn3219045, accessed on 1 August 2020) [50], consisting of transcriptomes of 146 MCI (mild cognitive impairment), 193 AD, and 189 region-matched control tissues. All libraries were prepared with the strand-specific RNA-seq paired-end protocol, and each, on average, consisted of 42.8484 million reads. The detailed information about these samples is given in Supplementary Table S1.

Enzymes and reactions: The balanced equation of the chemical reaction catalyzed by each enzyme encoded in the human genome, along with the number of H^+^ produced or consumed, was retrieved from the HumanCyc database [51].

### 4.2. Methods

#### 4.2.1. RNA-seq Processing and lncRNA Identification

High-quality clean reads were obtained by using the clipping adapters, and low-quality reads were identified using SOAPnuke [52]. Each library was individually mapped to the *GENCODE* genome (version 34) using HISAT2 (version 2.7) [53] with the default parameters. The resulting alignment files were used as input for transcript assembly using StingTie (version 2.1.1) [54], and the *Homo sapiens* reference annotation of GENCODE (release 34). All StringTie output files were merged into one unified transcriptome using the merge option of StringTie. Gffcompare [54] was employed to compare the resulting transcriptome (GTF format) with the reference annotation. The genome FASTA files and annotation files for the assembled transcripts are given in Supplementary Data S4. For consistency in nomenclature, we adopted StringTie’s default “MSTRG” as the starting point when naming all assembled transcripts. All new lncRNA sequences and annotation files, as well as all protein-coding transcripts, along with their corresponding gene names and chromosomal locations, are available in Supplementary Data S1.

#### 4.2.2. The Prediction Process for lncRNA Genes

Step 1: Screening for protein-coding potentials. Transcript lengths and the number of exons were collected in the basic screening step. Here, transcripts having lengths  >  200 nt and at least two exons were selected as lncRNA candidates [55]. The protein-coding potential of a transcript was predicted jointly using five widely used methods: CPC (Coding Potential Calculator) [56], CNCI (Coding-Non-Coding Index) [57], CPAT (Coding Potential Assessment Tool) [58], PLEK [59], and CPPred [60].

Step 2: A candidate is predicted to be an lncRNA if all five methods predict it to be an lncRNA. If the transcript was newly expressed (not in GENCODE), it was assigned one of the following codes: *i* (transcript contained entirely in an intron), *y* (containing a reference within its introns), *p* (not inside but within 2 Kb of a known transcript), or *u* (in an intergenic region). All lncRNAs were then classified into three types: intergenic lncRNAs, antisense lncRNAs, and intronic lncRNAs.

#### 4.2.3. Differential Analysis of All Assembled Transcripts

Differential expression analyses between the (MCI ⋃ AD) samples and controls were conducted using the “DESeq2” function [61]. For read-count data, transcripts with |FC| ≥ 1.3 having a statistical significance FDR < 0.05 were considered differentially expressed transcripts (DETs), where FC is for fold change. Transcripts with DESeq2 normalized read counts less than 1 will be excluded.

The TPM values of all genes from Ballgown [62] were used to assess correlations between lncRNAs and mRNAs using the Spearman correlation test, where correlation coefficients > 0 with adjusted *p*-values < 0.05 were used as the cutoffs for co-expressed transcript pairs. The expression levels of lncRNAs for all samples can be found in Supplementary Table S16.

#### 4.2.4. Ordering Disease Samples by the Level of Deviation from Control Tissues

A differential expression analysis was conducted on each sample in (MCI ⋃ AD) compared to all controls, yielding a differential intensity (V) value for each sample, defined as the sum of the absolute values of the differential expressions over all DETs with an adjusted *p*-value < 0.05. Intuitively, this value reflects the “distance” between a disease sample and the control samples in terms of their gene expression, giving rise to a possible way to approximate the progression of the disease. However, we noted that the order so defined was not very stable for different values of parameters {FC, *p*-value}, giving rise to different orderings of the samples when different values for {FC, *p*-value} were used. Hence, we conducted an analysis of the relationships between the different orderings of the disease samples and the quality of the approximation to the disease progression, using measures such as the increasing or decreasing trends of death rate, extracellular acidity, intracellular alkalinity, and a few others along the disease progression axis.

The result of the analysis indicated that the ordering that optimized the following empirical function achieved the best approximation to the disease progression based on the above consideration. Note that each set of values of {FC, *p*-value} gives rise to a distinct ordering of the samples and the associated {V_i_} values, where FC was selected from the range (1.1, 4.0) and *p*-value from (0.0005, 0.05), using 0.1 and 0.0001 as the increments for the former and the latter, respectively:

$$\underset{\mathrm{FC},p-\mathrm{value}}{\max}\sum_{1\leq i\leq \left|MCI\cup AD\right|}V_{i}$$

**Subject to** 1. The first |MCI| samples in the current order contain at least γ% of MCI samples. 2. The last (|MCI| + |AD|/2) samples contain at least γ% of AD samples, where |X| denotes the number of elements in set X, and γ is set to 55 based on our empirical analysis results.

The following provides a justification for why this list is useful for studies of AD progression, along with its applications. We note that (1) the levels of both intracellular alkalinization and extracellular acidification progressively go up with this approximation, as shown in Figure 3A, and (2) the same is observed about the levels of Aβ formation, Tau-fibril formation, oxidative stress, and neuronal apoptosis, as shown in Figure 3A, which are consistent with previous studies [1,2], thus supporting the validity of this approximation scheme to disease progression. Note that all the changes here were estimated based on the relevant marker genes established in the literature [63] or our own analyses (internal database).

For a given list of samples ordered as above, we partition it into four equal-sized sub-lists: bin1, bin2, bin3, and bin4 (possibly except for the last one), representing four periods of the disease evolution (Figure 3A). The specific bin assignment for each sample is provided in Supplementary Table S1. For the current study, the number of differentially expressed transcripts in the four bins was 9193, 8947, 10,279, and 20,496, respectively.

#### 4.2.5. ssGSEA Gene-Set Scores for Individual Samples

For gene-set enrichment analysis (GSEA) over individual samples, we employed the “gsva()” function in R, utilizing the “ssgsea” method, tailored specifically for GSEA on individual samples [64], which builds on an empirical cumulative distribution function (ECDF) of gene expression ranks. First, gene expression data were ranked across all samples. Then, for each specified gene set, the ECDF was computed based on the ranks of the genes within and outside the set. This comparison allowed the method to determine the extent to which genes within the gene set were enriched by the highly ranked genes. Positive enrichment scores signify an overrepresentation of the gene set among highly ranked genes, while negative scores indicate being enriched by lowly ranked genes.

#### 4.2.6. Co-Expression Analyses

The following is used to estimate the co-expression level between a gene g and a gene set M in terms of their expression levels. Let PCi represent the ith principal component of the expression levels of gene set M. It is required that the selected PCs can collectively explain at least 60% of the variance of the expression matrix. A linear regression model was constructed as below:

$$e\left(g\right)=\beta_{i}PC_{i}+\beta_{0}$$

where {$\beta_{i}$} are the parameters to be determined through minimizing |$\beta_{0}$|.

#### 4.2.7. Predicting Target Genes or Pathways of *cis* and *trans* Regulation by lncRNA

An lncRNA can participate in the regulation of expressions of genes in its genomic neighborhood, referred to as *cis*-regulation, or genes distant from its genomic location, called *trans*-regulation [65,66]. Prediction of target genes regulated by an lncRNA via the *cis* mechanism relies on the locational relationship. Genes within a range of 100 kb up- or downstream of an lncRNA are considered as potentially *cis*-regulated target genes by the lncRNA if they are co-expressed with the lncRNA [67,68]. *Trans*-regulated genes by an lncRNA are determined based on sequence complementarity between the lncRNA and each target gene and being co-expressed between the two. RIsearch [69] was used to predict trans-regulated genes by an lncRNA, where the prediction criteria also included the level of the free energy in the formed secondary structure between lncRNA and mRNA sequences (energy < −10) [70].

We further define the regulatory relationship between an lncRNA and pathways as follows: an lncRNA is considered a regulator of a pathway if an optimal regression model of the lncRNA against the principal components (PCs) of the pathway across all the relevant samples yields a high R^2^ value and an adjusted *p*-value < 0.05; if the lncRNA is predicted to be a *cis* or *trans* regulator of any gene (or transcript) in the pathway, with a Spearman correlation coefficient |r| > 0 and the adjusted *p*-value < 0.05 with the lncRNA strongly correlating with the first PC of the pathway.

#### 4.2.8. Construction of lncRNA-mRNA Co-Expression Network and Identification of Functional Modules

For a set of disease samples, we constructed an lncRNA–mRNA co-expression network based on co-expressed lncRNA-mRNA pairs using the method given in [71], for all mRNAs of the protein-coding genes. Specifically, for each pair of lncRNA and mRNA, we calculated the Spearman correlation coefficient (SCC) between the expressions of the pair across the specified samples. All pairs with |SCC| > 0 constitute the network, referred to as a *bi-color network*, following the definition in [72]. We then applied the Markov clustering algorithm [73] to identify maximal subnetworks having dense intra-interactions of the network, referred to as *clusters*.

For all the identified clusters, we sorted them by the descending order of the numbers of lncRNAs contained in each. We then calculated the cumulative number of lncRNAs within each bin and eliminated clusters until the cumulative number of lncRNAs reached 95% of the total number of lncRNAs. Pathway enrichment was conducted over each set of gene clusters against three databases: KEGG [74], REACTOME [75], and GO Biological Process [64] using Clusterprofiler [76] and an adjusted *p*-value < 0.05 as the threshold.

The enriched pathways were grouped into six categories: immune activities, metabolism, stress response, development-related, cell polarity, and neural functions. Here, we considered the glial cells, such as astrocytes and microglia, as immune cells.

The idea in defining the mRNA–lncRNA co-expression network and conducting the network-based clustering was to assign lncRNAs to pathways enriched by functionally well-annotated mRNAs, therefore giving some functional information to the lncRNAs.

#### 4.2.9. The Discerning Power of lncRNAs in Distinguishing Control from AD Samples

Considering three sample sets: $D_{1}=$ controls, $D_{2}=$ bin1, and $D_{3}=$ $\underset{2\leq i\leq 4}{⋃}bini$, for normal, early, advanced AD samples. Let their sizes be s_1_, s_2_ and s_3_, respectively, and D = $D_{1}\;\cup \;D_{2}\;\cup \;D_{3}$. For the three sample sets, each of their elements, d, has a label (d) *=* 1, 2, or 3 if it belongs to the first, second, or third set, respectively. We aimed to demonstrate that some lncRNA-containing pathways had the discerning power to separate the three sets.

Considering a pathway P enriched by genes {$g_{1},\;\ldots ,\;g_{s}\}\;$ and {$D_{1}$, $D_{2}$, $D_{3}\}$. We define the discerning power by P between sets $D_{1}$ and $D_{2}$ (defined similarly for $D_{1}$ and $D_{3}$) as follows.

Let $d_{1}^{i}$ = ($x_{i1}$, …, $x_{is}$) be the expressions of {$g_{1},\;\ldots ,\;g_{s}\}$ in the ith sample of $D_{1}$, and $d_{2}^{j}$ = ($y_{j1}$, …, $y_{js}$) be the expressions of {$g_{1},\;\ldots ,\;g_{s}\}$ in the jth sample of $D_{2}$. Our goal was to find a linear function **w:** $R^{s}\to$ R so that the distance between the average expressions of {$g_{1},\;\ldots ,\;g_{s}\}$ in $D_{1}$ and $D_{2}$ was as high as possible, and the dispersion within $D_{1}$ (and $D_{2})$ as small as possible, which can be formulated mathematically to find a **w:**

$$w=\;\underset{w}{\arg\;\max}\frac{w^{T}S_{B}w}{w^{T}S_{W}w}$$

where dispersion matrix within sets $S_{W}$ is defined as follows:

$$S_{W}=\sum_{i=1}^{m}(d_{1}^{i}-\mu_{1})(d_{1}^{i}-\mu_{1}{)}^{T}+\sum_{j=1}^{n}(d_{2}^{j}-\mu_{2})(d_{2}^{j}-\mu_{2}{)}^{T}$$

with m = $\left|D_{1}\right|$ and n = $\left|D_{2}\right|$, and $\mu_{i}$ is the expression vector of {$g_{1},\;\ldots ,\;g_{s}\}$ averaged over all samples in $D_{i}$; the dispersion matrix between the two sets $S_{B}$ is defined as follows:

$$S_{B}=(\mu_{1}-\;\mu_{2})\;(\mu_{1}-\mu_{2}{)}^{T}.$$

This problem can be solved through solving the following generalized eigenvalue problem:

$$S_{B}w=\lambda S_{W}w.$$

Let $w^{*}$ be an optimal solution to the above equation, and let $\mu_{i}^{*}$ and $s_{i}^{*}$ be the mean and the variance of the $w^{*}$-projected expressions of {$g_{1},\;\ldots ,\;g_{s}\}$ in $D_{i}$, respectively. Following the Fisher’s discrimination criterion, we defined the following:

$$F=\frac{{(\mu }_{1}^{*}-\mu_{2}^{*}\;{)}^{2}}{{(s}_{1}^{*}-s_{2}^{*}\;{)}^{2}}$$

To facilitate easy application, we defined the discerning score DS ∈ [0, 1.0] as follows:

$$\mathrm{DS}=\frac{F}{1+F}$$

Clearly, the higher the F is, the closer DS is to 1.0, and DS = 0 when P has no discerning power [77].

## Figures and Tables

**Table 1:** The number of lncRNAs with high discerning scores involved in immune activity, metabolic reprogramming, cell polarity, and stress response from bin1 through bin4.

Sub-List	Immune Activity	Metabolic Reprogramming	Cell Polarity	Stress Response
bin1	160	339	876	827
bin2	1069	729	1569	1291
bin3	863	1362	1522	1421
bin4	1916	5156	5230	5204

**Table 2:** AUC scores achieved by lncRNAs in distinguishing between all AD samples vs. controls, late-stage AD samples vs. controls, early-stage AD patients vs. controls, and early-stage vs. late-stage AD samples.

Phenotype	Normal_vs_allAD	Normal_vs_eAD	Normal_vs_aAD	eAD_vs_aAD
Aciding extrusion transporter	0.7124	0.6018	0.8998	0.9499
Aciding loading transporter	0.6448	0.6539	0.825	0.9602
Active oxygen	0.7297	0.7365	0.8673	0.8543
Astrocytes	0.6653	0.7124	0.8029	0.9718
Hydrogen peroxide	0.6981	0.851	0.8412	0.9339
Microglia	0.6669	0.581	0.8614	0.9429
Superoxide anion generation	0.7015	0.823	0.944	0.9416
Bicarbonate transporter	0.6819	0.6675	0.8595	0.9685
Extracellular acidosis	0.6373	0.6425	0.8783	0.878
Na^+^/K^+^-ATPase	0.7866	0.8256	0.8881	0.9615
Cholesterol	0.6694	0.6871	0.8939	0.9557
Extracellular copper	0.701	0.8783	0.8718	0.9018
Mitochondrial iron–sulfur	0.6378	0.5979	0.8022	0.8228
H-producing enzyme	0.6971	0.6662	0.8399	0.8652
Microtubule depolymerization	0.7562	0.8256	0.8198	0.9268
Synapse assembly	0.6909	0.7235	0.8979	0.8967
Amyloid formation	0.6914	0.6012	0.8855	0.9634
Tau fiber formation	0.6858	0.6291	0.8549	0.957

Note: Includes all AD-related cognitive impairment patients, i.e., Bin 1 to Bin 4; Early-stage cognitive impairment patients, i.e., Bin 1; Late-stage cognitive impairment patients, i.e., Bin 2, 3, and 4.

## Data Availability

The data supporting the reported results can be found at https://drive.google.com/drive/folders/1ORPZyNHtC0g74rZXQ9-2y6_55NVnvRaU?usp=sharing (accessed on 1 August 2024).

## Supplementary Materials

The following supporting information can be downloaded at https://www.mdpi.com/article/10.3390/ijms25179211/s1 and https://drive.google.com/drive/folders/1ORPZyNHtC0g74rZXQ9-2y6_55NVnvRaU?usp=sharing (accessed on 1 August 2024).
