# Supplementary material for: Elucidating the Functional Roles of Long Non-Coding RNAs in Alzheimer’s Disease
Source: Int J Mol Sci. 2024 Aug 25;25(17):9211. doi: 10.3390/ijms25179211 (PMC11394787; doi:10.3390/ijms25179211)
Supplement: Supplementary file 1 [file ijms-25-09211-s001.zip › ijms-3162723-supplementary.pdf]

SUPPLEMENTARY MATERIAL FOR

Elucidating the Functional Roles of Long Non-coding RNAs in Alzheimer's Disease

The PDF file includes:
Supplementary Tables: Table S1 to S16
Supplementary Data: Data S1 to S4
Supplementary Figures: Figure S1 to S5

A. **Supplementary Tables**

Table S1: Clinical information of ROSMAP prefrontal cortex samples.

Table S2: The four bin pathways contributed to the six categories of pathway statistics in the top 20%.

Table S3: Statistics of lncRNAs regulating microtubule depolymerization in cis and trans, along with mRNA enrichment results associated with lncRNAs regulating microtubule depolymerization.

Table S4: Statistics of lncRNAs regulating Tau fibrillization in cis and trans, along with mRNA enrichment results associated with lncRNAs regulating Tau fibrillization.

Table S5: Statistics of lncRNAs regulating amyloid formation in cis and trans, along with mRNA enrichment results associated with lncRNAs regulating amyloid formation.

Table S6: Statistics of lncRNAs regulating hydrogen peroxide in cis and trans, along with mRNA enrichment results associated with lncRNAs regulating hydrogen peroxide and superoxide anion generation.

Table S7: Statistics of lncRNAs regulating mitochondrial iron sulfur and copper transport in cis and trans, along with mRNA enrichment results associated with lncRNAs regulating mitochondrial iron sulfur.

Table S8: Statistics of lncRNAs regulating extracellular copper in cis and trans, along with mRNA enrichment results associated with lncRNAs regulating mitochondrial iron sulfur.

Table S9: Statistics of lncRNAs regulating synapse assembly in cis and trans, along with mRNA enrichment results associated with lncRNAs regulating synapse assembly.

Table S10: Statistics of lncRNAs regulating cholesterol in cis and trans, along with mRNA enrichment results associated with lncRNAs regulating cholesterol.

Table S11: Statistics of lncRNAs regulating H-producing enzyme in cis and trans, along with mRNA enrichment results associated with lncRNAs regulating H-producing enzyme.

Table S12: PCA analysis of H^+-producing enzymes reveals the ranking of each gene's contribution to PC1 explained variance ratio**.**

Table S13: Statistics of lncRNAs regulating aciding loading transporter in cis and trans, along with mRNA enrichment results associated with lncRNAs regulating aciding loading transporter.

Table S14: All gene sets regulated by lncRNAs.

Table S15: LncRNAs predicted as markers regulating each AD phenotype and cellular stress.

Table S16: lncRNA expression data for all samples

B. **Supplementary Data**

Supplementary Data S1: New lncRNA sequences and genome annotations, including the genomic
locations of all transcripts, and protein-coding transcripts with their corresponding gene names.

Supplementary Data S2: Pathway enrichment information for each MCL subclass under Bin1 to Bin4.

Supplementary Data S3: Gene sets of protein-coding genes involved in the AD system model.

Supplementary Data S4: New genome annotation files and all transcript sequences assembled by StringTie.

C. **Supplementary Figures**

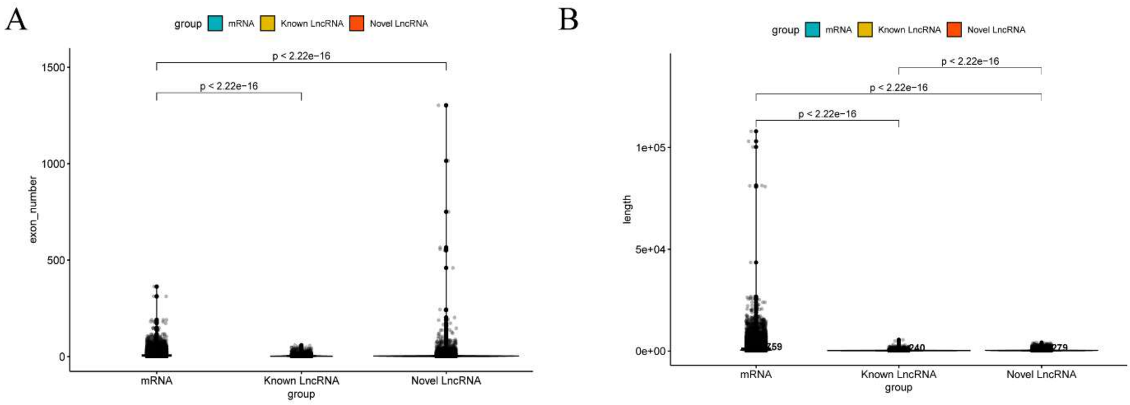

Figure S1. Summary of Exon Count and Length for mRNA, Old lncRNA, and New lncRNA. a. Statistics of exon counts for mRNA, old lncRNA, and new lncRNA. **b.** Statistics of RNA length for mRNA, old lncRNA, and new lncRNA.

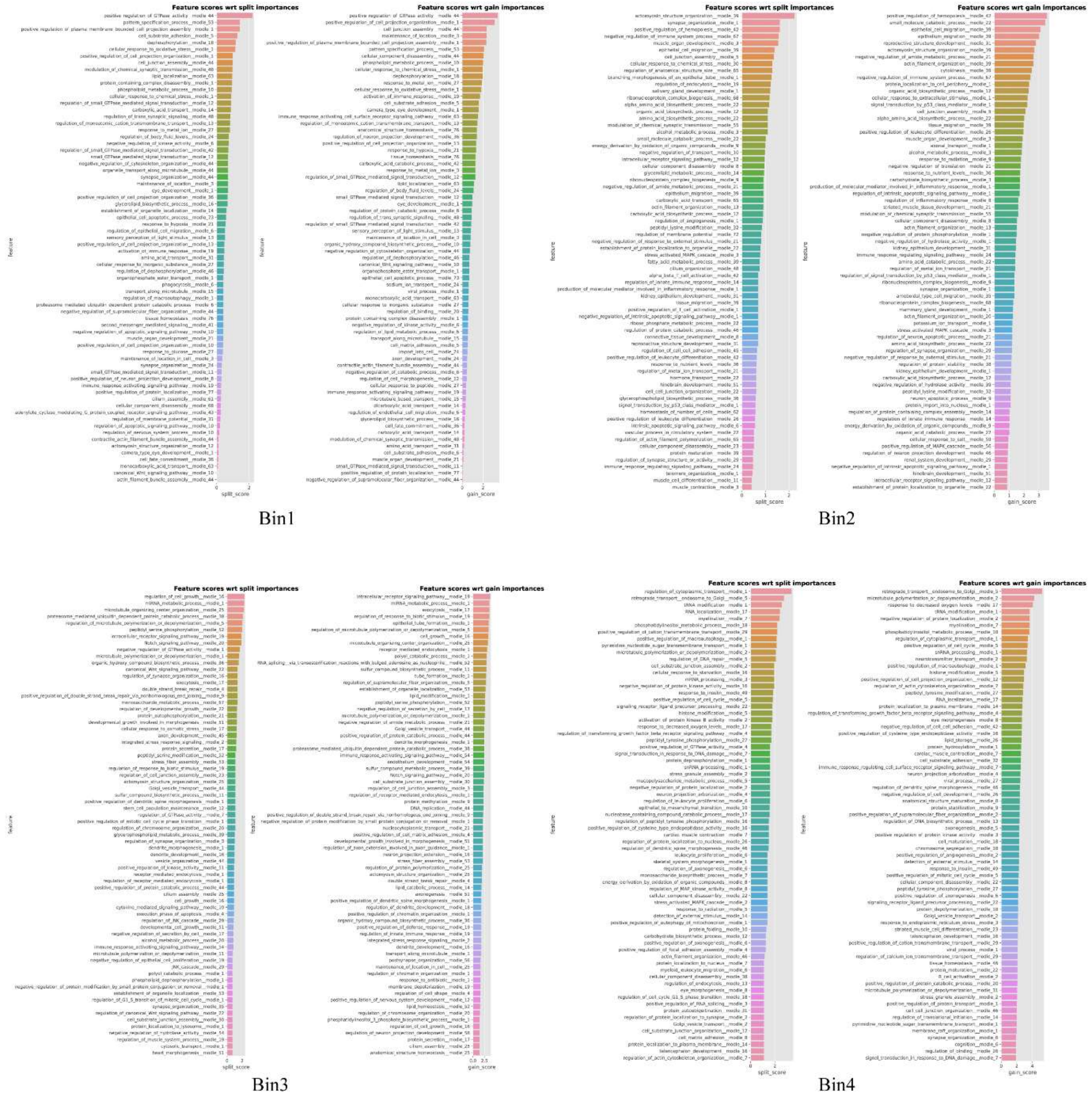

Figure S2. Evaluation of contribution of all four bin pathways to AD samples.

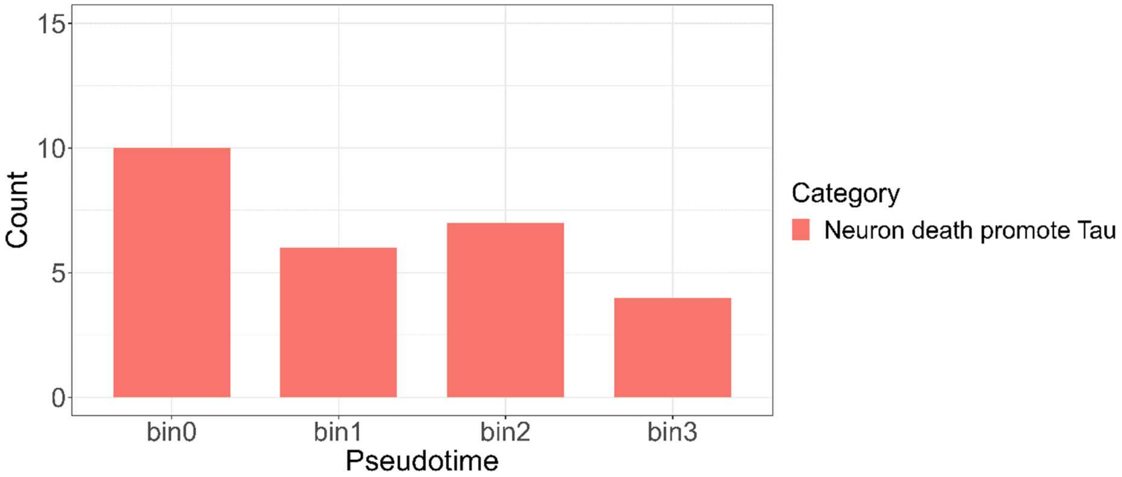

Figure S3. Number of neuronal apoptosis pathways and neuron death promoted by lncRNA in the formation of Tau fibers**.**

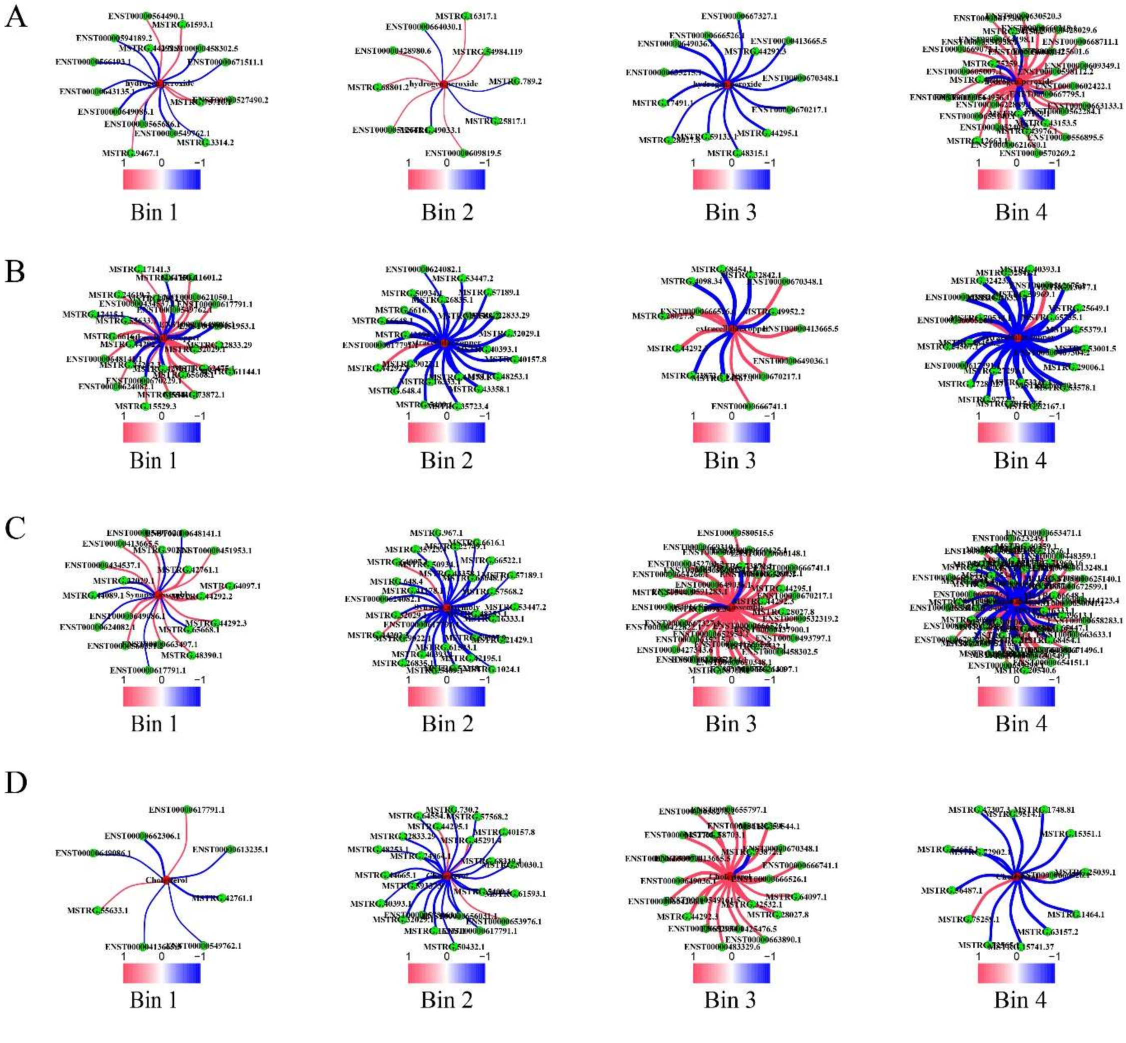

Figure S4. **Statistics of lncRNA associated with pathologic hypotheses of AD. A.** The regulatory trends of lncRNAs associated with hydrogen peroxide across the four bins. **B.** The regulatory trends of

lncRNAs associated with extracellular copper release across the four bins. **C.** The regulatory trends of lncRNAs associated with synapse assembly across the four bins. **D.** The regulatory trends of lncRNAs associated with cholesterol synthesis across the four bins.

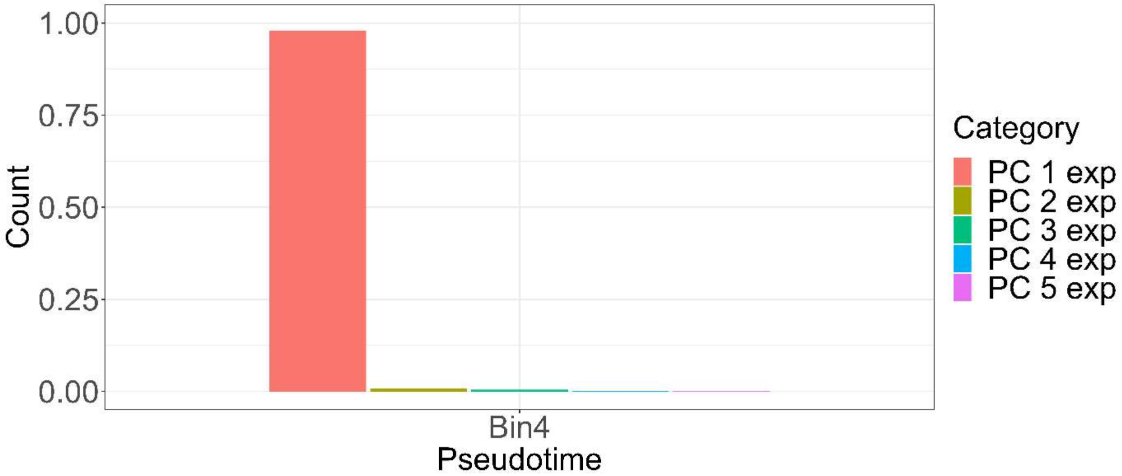

Figure S5. PCA contribution rate statistics of H^+-producing enzyme genes in late-stage AD (Bin4).
